# Predictive value of CpG island methylator phenotype for tumor recurrence in hepatitis B virus-associated hepatocellular carcinoma following liver transplantation

**DOI:** 10.1186/1471-2407-10-399

**Published:** 2010-08-02

**Authors:** Li-Ming Wu, Feng Zhang, Lin Zhou, Zhe Yang, Hai-Yang Xie, Shu-Sen Zheng

**Affiliations:** 1 Key Lab of Combined Multi-organ Transplantation, Ministry of Public Health. Key Lab of Organ Transplantation, Zhejiang Province, China and Department of Hepatobiliary and Pancreatic Surgery, the First Affiliated Hospital, Zhejiang University School of Medicine, Hangzhou, China

## Abstract

**Background:**

CpG island methylator phenotype (CIMP), in which multiple genes concordantly methylated, has been demonstrated to be associated with progression, recurrence, as well as overall survival in some types of cancer.

**Methods:**

We examined the promoter methylation status of seven genes including *P16, CDH1, GSTP1, DAPK, XAF1, SOCS1 *and *SYK *in 65 cases of HCC treated with LT by methylation-specific PCR. CIMP+ was defined as having three or more genes that are concordantly methylated. The relationship between CIMP status and clinicopathological parameters, as well as tumor recurrence was further analyzed.

**Results:**

CIMP+ was more frequent in HCC with AFP > 400 ng/ml than those with AFP ≤ 400 ng/ml (*P *= 0.017). In addition, patients with CIMP+ were prone to have multiple tumor numbers than those with CIMP- (*P *= 0.007). Patients with CIMP+ tumors had significantly worse recurrence-free survival (RFS) than patients with CIMP-tumors by Kaplan-Meier estimates (*P *= 0.004). Multivariate analysis also revealed that CIMP status might be a novel independent prognostic factor of RFS for HCC patients treated with LT (HR: 3.581; 95% CI: 1.473-8.710, *P *= 0.005).

**Conclusion:**

Our results suggested that CIMP could serve as a new prognostic biomarker to predict the risk of tumor recurrence in HCC after transplantation.

## Background

Primary liver cancer is one of the most common solid tumors, rated fifth in incidence and the third in mortality worldwide [1]. Hepatocellular carcinoma (HCC) accounts for between 85% and 90% of primary liver cancers [2]. China is one of the highest prevalent areas of HCC, mainly because of chronic hepatitis B carriers accounting for more than 10% of its population [3]. The prognosis of patients with HCC remains generally poor, even after surgical resection or chemotherapy. Liver transplantation (LT) offers a potential curative option for patients with small HCC, but post-operative tumor recurrence remains one of the most prevalent causes of unsatisfactory long-term survival [4]. Therefore, identification of reliable prognostic factors for tumor recurrence and death could have significant clinical importance. Patients in a low-risk group, for example, would be more appropriated candidates for LT, which is benefit for establishing a new set of election and prognostic criteria.

Over the past few years, both our group and others have focused on searching for reliable molecular biomarkers to better distinguish subtypes of patients who have different risk of tumor recurrence in HCC patients treated with LT [5-7]. Investigators in our group have established a retrospective cohort of HCC patients who underwent LT at our institution, and analyzed some potential tumor biomarkers within this valuable clinical research database. Yet little is known about the epigenetic biomarkers for selection and prognostic prediction after LT.

Recently, as an important mechanism of inactivation of tumor suppressor genes (TSGs), DNA methylation has shown promise as a potential biomarker for early detection, therapy monitoring, assessment of prognosis or prediction of therapy response in a variety of malignancies [8-11], including HCC [12,13]. However, in recent years, a methylator phenotype based on concurrently methylated of multiple TSGs, also called the CpG island methylator phenotype (CIMP), is being considered to have more clinical value than a single gene methylation. [14]. Numerous studies have suggested that CIMP status might be associated with progression, recurrence, as well as long-term survival in different types of cancer, such as non-small cell lung cancer (NSCLC) [15], acute lymphoblastic leukemia [16], neuroblastoma [17], esophageal adenocarcinoma [18]and colon cancer [19]. In HCC, Zhang et al. [20] detected a panel of CIMP including nine TSGs in 50 HCC patients with surgical resection, and found that CIMP status was correlated with elevated preoperative serum AFP level. More recently, Cheng et al. [21] examined the promoter methylation status of 10 genes in 60 cases of HCC with surgical resection, and the results suggested that CIMP could serve as a molecular marker of late stage and poorly prognostic HCC development. However, the predictive value of CIMP for tumor recurrence in HCC patients, especially in HCC treated with LT, remains unclear. Therefore, it is worthy of developing a panel consist of representative genes from key molecular pathways or a selection reflecting the CIMP status of HCC patients treated with LT.

In this study, in order to investigate the predictive value of the methylation status of a panel of TSGs on tumor recurrence in HCC, the promoter methylation of twelve TSGs that belonging to the molecular pathways involved in cell immortalization and transformation included *P16, CDH1, GSTP1, DAPK, MGMT, XAF1, TIMP3, SOCS1, SFRP1, TMS1, SYK *and *DKK1 *were initially examined in a small cohort of 20 cases of HCC treated with LT [12,22-31] (Table 1). These genes were selected because they have been demonstrated to be methylated frequently in HCC and other malignancies. Resultantly, seven target genes with methylation frequency more than 40% were brought to the panel of CIMP (including *P16, CDH1, GSTP1, DAPK, XAF1, SOCS1 *and *SYK*). Then the examination of methylation status of these seven individual genes was expanded to total 65 cases of hepatitis B virus (HBV)-associated HCC treated with LT. The relationship between aberrant methylation pattern of multiple genes and clinicopathological parameters, as well as tumor recurrence was further analyzed in this study. The aim of the present study was to determine whether CIMP is a potentially predictive biomarker of tumor recurrence in HCC patients following LT.

**Table 1:** Genes investigated for methylation in HCC after LT

Gene	Chromosomal Locations	Function	References
*SFRP1*	8p12-11.1	Wnt signaling pathway antagonist	[30]
*P16*	9q21	Cell cycle regulation	[22]
*SYK*	9q22	Signal transduction	[12]
*DAPK*	9q34	Interferon-γ, TNF-α, and FAS-induced apoptosis	[25]
			
*DKK1*	10q11.1	Wnt signaling pathway antagonist	[31]
*MGMT*	10q26	DNA repair	[26]
*GSTP1*	11q13	Carcinogens and cytotoxic drug detoxification	[24]
*TMS1*	16p11-12	Apoptosis regulation	[25]
*SOCS1*	16p13.13	Regulator of cytokine signaling	[29]
*CDH1*	16q22	Cell adhesion	[23]
*XAF1*	17p13.2	Cell apoptosis	[27]
*TIMP3*	22q13.1	Tissue invasion and metastasis	[21]

## Methods

### Patients and specimens

A total of sixty-five patients (59 men, 6 women; mean age 48.8 years; range, 29-67 years) who underwent LT in our institution between 2003 and 2005 were enrolled in this retrospective study according to the same eligibility criteria as our recent study [5]: (a) The diagnosis of HCC were confirmed by histopathologic examination either before or after transplantation (as an incidental finding); (b) all patients included in this retrospective study were HBV-positive; (c) The clinical and laboratory data was obtained for all 65 patients, including portal vein tumor thrombi (PVTT), preoperative alpha-fetoprotein (AFP) level, histopathologic grading, tumor size, and tumor number; (d) all these patients were Han Chinese; (e) none of these patients received preoperative adjuvant antineoplastic therapy. The follow-up course and diagnosis criteria of recurrence have been shown in the previous study [7]. This study was approved by local ethic committee, and informed consent was obtained according to the Declaration of Helsinki. All the tissue samples from primary tumors were stored at -80°C tissue banks.

### Methylation-specific polymerase chain reaction (MSP)

Aberrant promoter methylation of these genes was determined by method of methylation-specific polymerase chain reaction (MSP) as reported by Herman et al [32]. Genomic DNA was isolated from frozen liver tissue using DNeasy Tissue Kit (Qiagen, Valencia, CA) according to the manufacturer's instructions. DNA samples were modified using EZ DNA Methylation Golden Kit (Zymo Research, Orange, CA). MSP distinguishes unmethylated alleles of a given gene based on DNA sequence alterations after bisulfite treatment of DNA, which converts unmethylated but not methylated cytosines to uracils. Subsequent polymerase chain reaction using primers specific to sequences corresponding to either methylated or unmethylated DNA sequences was then performed. DNA methylation of CpG islands was then determined by PCR using specific primers for either methylated or unmethylated DNA. Two sets of primers were used to amplify each region of interest: one pair recognized a sequence in which CpG sites were unmethylated (bisulfite-modified to UpG), and the other recognized a sequence in which CpG sites were methylated (unmodified by bisulfite treatment). The MSP primer sequences of each gene for the unmethylated and methylated reactions were determined as described previously [12,22-31]. The PCR amplifications were carried out with treated DNA as template in a total volume of 25 μl containing 25 pM of each primers, 25 μM deoxynucleoside triphosphates, 30 ng of bisulfate-treated DNA, 1 U of hot-start Taq polymerase (Takara, Shiga, Japan) and the respective buffers. Hot start polymerase chain reaction was performed at 95°C hot start for 10 minutes followed by 30 repetitive cycles consisting of denaturation at 95°C for 30 seconds, annealing at specific temperature for 30 seconds, and extension at 72°C for 30 seconds, then finished with a final 10-minute extension. To prepare the positive methylation control, 1 μg of genomic DNA from normal human liver was treated in vitro with SssI methyltransferase (NEB, Beverly, MA), yielding completely methylated DNA at all CpG rich regions. Bisulfite-modified DNA from normal human liver served as a positive control for the unmethylated alleles. Water blanks were also included with each assay. The PCR products were analyzed on ethidium bromide-stained 2% agarose gel. No clinicopathological or follow-up data were revealed to the bench researchers until the MSP results were finalized.

### Statistical analysis

Statistical comparisons were performed using either the Pearson's chi-square test or two sided Fisher's exact test, as appropriate. Recurrence-free survival (RFS) was defined as the interval from the operation day to date of documented tumor recurrence or date of the most recent follow-up visit if recurrence did not occur. Log-rank tests were used to identify the number of methylated genes that was most predictive of RFS. Survival curves of the patients were compared using the Kaplan-Meier method and analyzed using the Log-rank test. For a given gene, a univariate Cox proportional hazard regression model was used to estimate the effect of methylation status (partially or completely methylated versus unmethylated) on the relative hazard of risk for tumor recurrence. The same method was also used to evaluate the univariate correlations between CIMP status or clinicalpathological variables, and tumor recurrence. Only variables with significance in univariate analysis were performed in the multivariate analysis using Cox proportional hazard model.

Data analysis was performed using Statistical Package for Social Sciences (SPSS) version 16.0 for Windows (SPSS Inc, Chicago, IL). All tests were two tailed and *P *< 0.05 was considered statistically significant.

## Results

### Frequency of CpG island hypermethylation in HCC

**Figure 1:**
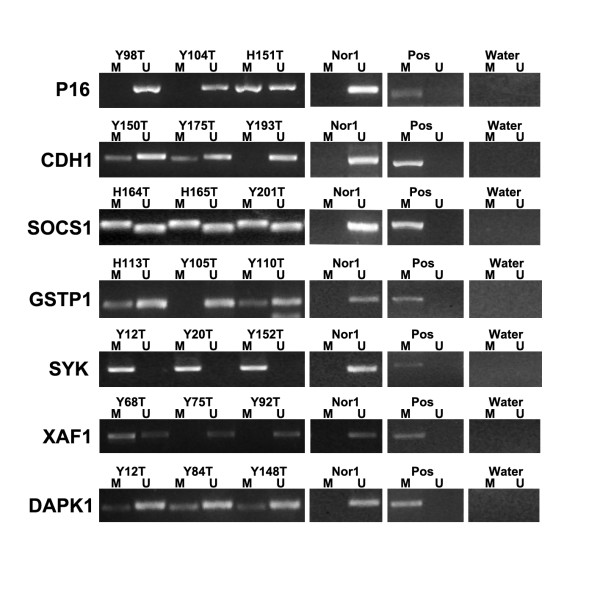
**Representative results of MSP for seven tumor suppressor genes**.

The frequency of promoter methylated of the gene included in the panel was 51% (33 of 65) for *P16*, 43% (28 of 65) for *CDH1*, 55% (36 of 65) for *SOCS1*, 60% (39 of 65) for *GSTP1*, 52% (34 of 65) for *SYK*, 66% (43 of 65) for *XAF1*, 52% (34 of 65) for *DAPK1*. The other five genes with the methylation frequency less than 40% were not analyzed in the CIMP panel (5% for *MGMT*, 15% for *TMS1*, 20% for *SFRP1*, 25% for *TIMP3*, 15% for *DKK1*). Representative examples of MSP assay results are presented in Fig. 1. Overall, only two cases showed no methylation of any of these genes.

Study of correlations between promoter methylation status of each gene and clinicopathological characteristics are summarized in Additional file 1. Overall, the hypermethylation of *GSTP1 *was present more frequently in patients with poor histopathologic grading (*P *= 0.003) and multiple tumor numbers (*P *= 0.011). The hypermethylaton of *XAF1 *was present more frequently in HCC, with AFP > 400 ng/ml than those with AFP ≤ 400 ng/ml (*P *= 0.007). Likewise, patients with methylated *DAPK *were suffered from larger tumor size than those with unmethylated *DAPK *promoter (*P *= 0.016). However, we found no significant associations between the methylation status of *P16*, *CDH1*, *SOCS1*, *SYK *and the following clinicopathological features: patient's age and gender, PVTT, AFP level, histopathologic grading, tumor size and tumor numbers.

The relative risk for tumor recurrence of methylation status of an individual gene was shown in Additional file 2. Only methylaton of *SYK *was found to be prone to recurrence (P = 0.046), which was similar to the previous study in HCC [12]. No significant correlation was observed between tumor recurrence and methylation status of any other individual gene.

### Frequency of CIMP in HCC patients

Originally, CIMP-positive gastric cancer was defined as a tumor with methylation at more than three genes [33]. CIMP was also defined by the average number of methylated genes per tumor in several recent studies [34,35]. However, a recent new study offered potential markers to define a CIMP concept, in which the thredshold distinguishing CIMP+ from CIMP-samples was chosen by minimizing the within-group sum of squared errors [36]. In the present study, a methylation phenotype was defined on the basis of results of log-rank tests of the effect of number of methylated genes on RFS as reported by Yang [37]. The lowest *P *value in Table 2 was used to identify an optimal cutoff of the number of methylated genes. Accordingly, CIMP status was classified as CIMP+ samples (with three or more methylated genes) and CIMP-samples (with two or fewer methylated genes). Of the HCCs, 72% (47 of 65) were CIMP+.

**Table 2:** Results of Log-rank tests for effect of number of methylated genes on recurrence-free survival in 65 HCC patients after LT

No. methylated genes	No. patients with profile in total 27 recurrence-free patients	No. patients with profile in total 38 recurrence patients	P*
≥1	25	38	0.152
≥2	22	36	0.107
≥3	15	32	0.004
≥4	14	29	0.015
≥5	8	16	0.478
≥6	2	6	0.280
≥7	0	2	0.236

HCC, hepatocellular carcinoma; LT, liver transplantation.

*Log-rank test

### Correlation of CIMP with clinicopathological parameters

Using statistical analysis, we examined CIMP with regard to HCC patient clinicopathologic parameters (Table 3). A significant difference between CIMP status and preoperative AFP level was being found in HCC with LT (*P *= 0.017). Meanwhile, we found that CIMP+ was more frequent in HCC, with AFP > 400 ng/ml (87%, 26/30) than those with AFP ≤ 400 ng/ml (60%, 21/35; *P *= 0.017). Likewise, patients with CIMP+ were prone to have multiple tumor numbers than those with CIMP- (*P *= 0.007). However, we found no significant associations between the CIMP status with other clinicopathological features like patient's age and gender, PVTT, histopathologic grading and tumor size. In addition, the results showed a correlation between CIMP and Hangzhou criteria, which consider tumor size, histological grading, and serum AFP level [38]. Patients that exceeded the Hangzhou criteria had a higher CIMP+ frequency compare to those matched Hangzhou criteria (84% vs. 57%, *P *= 0.017) (Table 3).

**Table 3:** Association of CIMP with clinicopathological parameters

Variables	Grading	CIMP		P*
		negative n (%)	positive n (%)	
Age(Years)	≤50	8(22)	28(78)	
	> 50	10(34)	19(66)	0.272
Gender	Female	2(33)	4(67)	
	Male	16(27)	43(73)	0.666
PVTT	Negative	13(30)	31(70)	
	Positive	5(24)	16(76)	0.629
Preoperative AFP level(ng/ml)	≤400	14(40)	21(60)	
	> 400	4(13)	26(87)	0.017
Histopathologic grading	Well+moderate	14(30)	33(70)	
	Poor	4(22)	14(78)	0.758
Tumor size(cm)	≤5	8(28)	21(72)	
	> 5	10(28)	26(72)	0.986
Tumor number	Single	11(48)	12(52)	
	Multiple	7(17)	35(83)	0.007
Hangzhou criteria**	Yes	12(43)	16(57)	
	No	6(16)	31(84)	0.017

PVTT, portal vein tumor thrombi; AFP, alpha-fetoprotein

** Apart from the presence of macrovascular invasion, Hangzhou criteria are matched if total tumor diameter ≤ 8 cm, or if, total tumor diameter > 8 cm, with histopathologic grade I/II and AFP ≤ 400 ng/mL [38].

*Pearson's Chi-square test or Fisher's exact test.

### CIMP correlates with the risk of tumor recurrence

**Figure 2:**
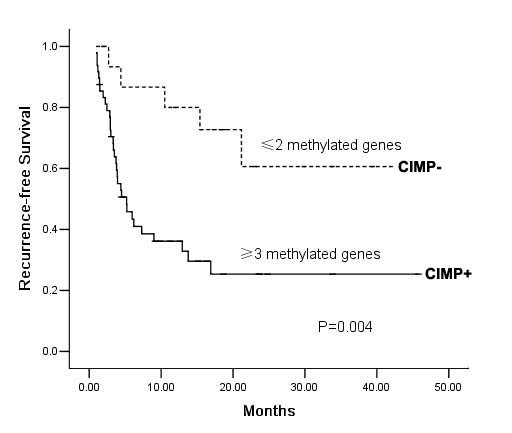
**Kaplan-Meier estimates of cumulative RFS in the 65 hepatocellular carcinoma patients treated with liver transplantation according to the CIMP status**. CIMP status was classified as CIMP+ samples (with three or more methylated genes) and CIMP- samples (with two or fewer methylated genes).

Recently, we used the Hangzhou criteria as a new criteria for prognosis prediction of HCC patients post-LT, and found those matched Hangzhou criteria had better RFS than the others. In this study, when the 65 patients were grouped according to the Hangzhou criteria, similar results were observed (*P *< 0.001, data not shown). Consistently, the cumulative 3-year RFS rate in patients with CIMP- is 64%, compared to that of 25% in patients with CIMP+. The mean RFS for CIMP- patients was significantly longer than that for CIMP+ patients by Kaplan-Meier estimates (30.3 vs. 15.3 months, *P *= 0.004) (Fig. 2). Considering the association of CIMP and Hangzhou criteria, the prognostic value of CIMP might overlap with that of Hangzhou criteria. Therefore, further segregation based on CIMP was applied to analyze the RFS in those who matched the Hangzhou criteria or not, respectively. Resultantly, a trend toward shorter RFS in CIMP+ than CIMP- was seen in both patients who matched the Hangzhou criteria and those exceeded the criteria, although there was no significant difference(*P *= 0.101,0.113, respectively, data not shown). Furthermore, Cox univariate analysis also revealed that the clinicopathological variables could provide significant predictive values for recurrence including preoperative AFP level, tumor size and CIMP status (data not shown). Multivariate analysis revealed that a larger tumor size and CIMP+ could be independent prognostic factors for RFS (Table 4).

**Table 4:** Multivariate Cox regression analysis of variables related to tumor recurrence at univariate analysis

Variable	Relative risk of recurrence (95% CI)	P*
Tumor size		
> 5 cm vs. ≤5 cm	2.366 (1.186-4.720)	0.015
CIMP		
positive vs. negative	3.581 (1.473-8.710)	0.005

*95% CIs and P values were calculated by the Cox proportional hazards regression analysis.

## Discussion

Recently, limited studies have confirmed the relationship between CIMP status and the progression, as well as prognosis of HCC with resection [20]. In this study, we also found that the CIMP status of patients with HCC after LT was closely associated with clinicopathological parameters, such as preoperative AFP level and tumor number. Furthermore, patients with CIMP+ tumors had significantly shorter RFS than patients with CIMP- tumors. To our best knowledge, this is the first investigation to develop a marker panel for improved recurrence prediction in HCC patients treated with LT.

The significance of CIMP-positive phenotype that is associated with distinct clinicopathologic features, such as advanced tumor stage and poor differentiation, have been best characterized in several types of malignancies [10,11]. In HCC, Zhang et al detected the promoter methylation status of nine genes in 50 pairs of HCC cases, and reported that patients with CIMP + had an elevated serum AFP level compare to that of CIMP- [20]. In line with this finding, we found that CIMP+ was more frequent in HCC with AFP > 400 ng/ml than those with AFP ≤ 400 ng/ml (*P *= 0.017). Further studies to clarify the relationship between AFP expression and methylator phenotype in HCC progress are recommended. Meanwhile, our results revealed that patients with CIMP + displayed a higher frequency of tumor multifocality than those with CIMP- (*P *= 0.007) (Table 3), suggesting a strong association between CIMP status and tumor numbers of HCC. Taken together, these findings further support that CIMP is a marker reflecting clinicopathologic features of human solid tumors involving HCC, suggesting the potential importance of CIMP in cancer development and prognosis. Based on the association between CIMP status and clinicopathological parameters such as AFP level and tumor numbers, we speculated that the CIMP might be correlated to Hangzhou criteria, which is a new criteria for patient selection and prognosis prediction of LT in HCC patients [38]. Obviously, the present data demonstrated this possibility. These results suggest that both CIMP and Hangzhou criteria have the power to reflecting the clinical and pathological factors of HCC. Possible, CIMP status might be an epigenetic molecular model of Hangzhou Criteria and have the similar potential ability to predict tumor recurrence after LT.

The prognostic role of CIMP status in HCC is unclear and there is only limited information about the prognostic significance of concordant gene methylation. A recent report in 60 HCCs with surgical resection found that metastasis was significantly different among patients with different CIMP status [21]. Furthermore, patients with high frequency CIMP tumors had significantly worse survival than patients with intermediate frequency or no CIMP tumors. These findings led us to investigate the potential correlation of CIMP status and the risk of tumor recurrence after LT. Similar to above finding, we found that patients with CIMP- showed favorable RFS than those with CIMP + (*P *= 0.004, Fig. 2). In order to further assess the independent impact of the different parameters on tumor recurrence, CIMP status as well as other parameters with significant association in the univariate analysis were included in the multivariate model. Analysis of data showed patients with CIMP + conferred a 3.6-fold increase in recurrence risk, which suggested that CIMP status might be a novel independent factor affecting tumor recurrence after LT (Table 4). Here, it should be mentioned that there was one minor limitation in this study. Although macrovascular invasion, tumor size, preoperative AFP level, and histopathologic grading have been proved to be independent prognostic factors for HCC patients following LT in most previous studies [6,7], Cox univariate analysis revealed that the clinicopathological variables that could provide significant predictive value for tumor recurrence only included preoperative AFP level, tumor size and CIMP status in the present study. We do not yet have a clear explanation for this result, but this is thought to at least in part due to the small sample size. Despite this limitation, these above findings suggested a major ability for CIMP in tumor invasiveness of HCC. In CIMP+ tumors, hypermethylated tumor suppressor-metastasis genes become simultaneously silenced. Consequently, it contributes to promoting proliferation and metastasis of tumor cells, which may subsequently result in increased risk of recurrence in patients with CIMP +. Given the prognostic power of CIMP status, further investigations are clearly warranted for a comparison of predictive value between Hangzhou criteria or Milan criteria and the CIMP marker in additional retrospective and prospective cohorts.

The term CIMP has been used for a subset of tumors with promoter methylation in multiple genes in many tumors. However, it should be concerned that there is no uniform selection pattern of CIMP in HCC up to now. The main reasons include three points: First, as known to all, methylation frequency of a certain gene varies among HCC patients due to the difference of ethnic origin, etiology background, and disease stages. In China, nearly 40% donor livers are offered to HCC patients, who have more hepatitis B related backgrounds and more advanced or aggressive tumor characteristics than those in Western countries [39]. Thus, it is almost impossible to establish a same CIMP panel as Western countries. Secondly, unlike in colon cancer, no systemic evaluation of a large, population-based sample strongly supports the biologic role of CIMP in HCC so far. Recently, Weisenberger et al. [36] conducted a systematic, stepwise screen of large-scale CpG island methylation markers using MethyLight technology in colon cancer and determined relatively uniform CIMP pattern. It would be worthwhile to develop a panel of representative genes involved key molecular pathways in HCC patients treated with LT using large-scale screen technology. In the present study, CIMP status was classified as CIMP+ samples (with three or more methylated genes) and CIMP- samples (with two or fewer methylated genes). And the mean RFS for CIMP- patients was significantly longer than that for CIMP+ patients (Fig. 2). These findings provided a valuable exploration for the predictive value of CIMP panel on tumor recurrence in HCC patients following LT. The study should therefore be viewed as hypothesis generating, to be followed by larger prospective and multiethnic studies to confirm our findings.

## Conclusion

In conclusion, Patients with CIMP+ had higher AFP level, multiple tumor numbers and unfavourable outcome. Furthermore, our study was the first one to establish the CIMP model as an independent marker to predict the risk of tumor recurrence in HCC after transplantation. Future studies will be focused on combining the CIMP pattern with Hangzhou criteria, and proposing a more reasonable and optimized model for patient selection and prognosis prediction, which will benefit more HCC patients for LT.

## Competing interests

The authors declare that they have no competing interests.

## Authors' contributions

LMW and FZ performed specimen collection, MSP, statistical analyses and wrote the manuscript. ZY contributed to the manuscript preparation. SSZ conceived the study and critically revised the manuscript. LZ and HYX supplied administrative support and revised the manuscript. All authors read and approved the final manuscript.

## Pre-publication history

The pre-publication history for this paper can be accessed here:

http://www.biomedcentral.com/1471-2407/10/399/prepub

## Supplementary Material

Additional file 1 **Supplementary Table 1**. Correlation between methylation status of each of the seven genes investigated and clinicopathological characteristics in HCC. Click here for file

Additional file 2 **Supplementary Table 2**. Results of Cox regression model for methylation status (partially or completely methylated versus unmethylated) of each separate gene on the relative risk for tumor recurrence. Click here for file
