# Supplementary material for: Predictive value of CpG island methylator phenotype for tumor recurrence in hepatitis B virus-associated hepatocellular carcinoma following liver transplantation
Source: BMC Cancer. 2010 Aug 2;10:399. doi: 10.1186/1471-2407-10-399 (PMC2922195; doi:10.1186/1471-2407-10-399)
Supplement: Additional file 1 — Supplementary Table 1. Correlation between methylation status of each of the seven genes investigated and clinicopathological characteristics in HCC. [file 1471-2407-10-399-S1.DOC]

**Additional file 1.**

Correlation between methylation status of each of the seven genes investigated and clinicopathological characteristics in HCC

|  | **No. of cases** | **Frequency of hypermethylation (%)** | | | | | | |
| --- | --- | --- | --- | --- | --- | --- | --- | --- |
|  |  | ***P16*** | ***CDH1*** | ***SOCS1*** | ***GSTP1*** | ***SYK*** | ***XAF1*** | ***DAPK*** |
| **Age(Years)** | | | | | | | | |
| **≤50** | 36 | 20(56) | 13(36) | 21(58) | 23(64) | 18(50) | 27(75) | 16(44) |
| **＞50** | 29 | 13(45) | 14(48) | 15(52) | 15(52) | 16(55) | 16(55) | 18(62) |
| **Gender** | | | | | | | | |
| **Female** | 6 | 3(50) | 2(33) | 2(33) | 2(33) | 3(50) | 5(83) | 2(33) |
| **Male** | 59 | 30(51) | 25(42) | 34(58) | 36(61) | 31(53) | 38(64) | 32(54) |
| **PVTT** | | | | | | | | |
| **Negative** | 44 | 23(52) | 20(45) | 21(48) | 22(50) | 25(57) | 29(66) | 23(52) |
| **Positive** | 21 | 10(48) | 7(33) | 15(71) | 16(76) | 9(43) | 14(67) | 11(52) |
| **Preoperative AFP level (ng/ml）** | | | | | | | | |
| **≤400** | 35 | 16(46) | 16(46) | 16(46) | 19(54) | 17(49) | 18(51)* | 17(49) |
| **＞400** | 30 | 17(57) | 11(37) | 20(67) | 19(63) | 17(57) | 25(83)* | 17(57) |
| **Histopathologic grading** | | | | | | | | |
| **Well+moderate** | 47 | 24(51) | 20(43) | 27(57) | 22(47)* | 24(51) | 30(64) | 24(51) |
| **Poor** | 18 | 9(50) | 7(39) | 9(50) | 16(89)* | 10(56) | 13(72) | 10(56) |
| **Tumor size（cm）** | | | | | | | | |
| **≤5** | 29 | 13(45) | 15(52) | 14(48) | 14(48) | 15(52) | 16(55) | 20(69)* |
| **＞5** | 36 | 20(56) | 12(33) | 22(61) | 24(67) | 19(53) | 27(75) | 14(39)* |
| **Tumor number** | | | | | | | | |
| **Single** | 23 | 8(35) | 10(43) | 11(48) | 8(35)* | 9(39) | 13(57) | 13(57) |
| **Multiple** | 42 | 25(60) | 17(40) | 25(60) | 30(71)* | 25(60) | 30(71) | 21(50) |

PVTT, portal vein tumor thrombi; AFP, alpha-fetoprotein.

**P*<0.05.
