# Supplementary material for: Predictive value of CpG island methylator phenotype for tumor recurrence in hepatitis B virus-associated hepatocellular carcinoma following liver transplantation
Source: BMC Cancer. 2010 Aug 2;10:399. doi: 10.1186/1471-2407-10-399 (PMC2922195; doi:10.1186/1471-2407-10-399)
Supplement: Additional file 2 — Supplementary Table 2. Results of Cox regression model for methylation status (partially or completely methylated versus unmethylated) of each separate gene on the relative risk for tumor recurrence. [file 1471-2407-10-399-S2.DOC]

**Additional file 2.** Results of Cox regression model for methylation status (partially or completely methylated versus unmethylated) of each separate gene on the relative risk for tumor recurrence

| Gene | Recurrence-free n (%) | Recurrence n (%) | HR(95%CI) | P* |
| --- | --- | --- | --- | --- |
| *P16* | 13/27(48) | 20/38(53) | 1.347(0.702-2.583) | 0.370 |
| *CDH1* | 12/27(44) | 15/38(39) | 0.819(0.426-1.577) | 0.551 |
| *SOCS1* | 11/27(41) | 25/38(66) | 1.956(0.999-3.830) | 0.050 |
| *GSTP1* | 13/27(48) | 26/38(68) | 1.707(0.856-3.404) | 0.129 |
| *SYK* | 11/27(41) | 23/38(61) | 1.995(1.014-3.926) | 0.046 |
| *XAF1* | 16/27(59) | 27/38(71) | 1.388(0.685-2.813) | 0.363 |
| *DAPK1* | 11/27(41) | 23/38(61) | 1.740(0.905-3.346) | 0.097 |

* Cox regression univariate analysis
